# Genetics against race: Science, politics and affirmative action in Brazil

**DOI:** 10.1177/0306312715610217

**Published:** 2015-12

**Authors:** Michael Kent, Peter Wade

**Affiliations:** Social Anthropology, The University of Manchester, Manchester, UK

**Keywords:** affirmative action, Brazil, genetics, politics, race

## Abstract

This article analyses interrelations between genetic ancestry research, political conflict and social identity. It focuses on the debate on race-based affirmative action policies, which have been implemented in Brazil since the turn of the century. Genetic evidence of high levels of admixture in the Brazilian population has become a key element of arguments that question the validity of the category of race for the development of public policies. In response, members of Brazil’s black movement have dismissed the relevance of genetics by arguing, first, that in Brazil race functions as a social – rather than a biological – category, and, second, that racial classification and discrimination in this country are based on appearance, rather than on genotype. This article highlights the importance of power relations and political interests in shaping public engagements with genetic research and their social consequences.

In May 2007, media coverage exploded in Brazil around the genetic ancestry of Neguinho da Beija-Flor. With his dark skin – Neguinho can be roughly translated as ‘blackie’ – and his leading role in Rio de Janeiro’s famous Beija-Flor samba school, he is a highly visible symbol of the country’s black community and culture. Neguinho’s DNA was tested as part of the Afro-Brazilian Roots project, commissioned by BBC Brazil, which investigated the genetic profiles of nine black celebrities – with a particular focus on their African ancestry – in order to raise the interest of the Brazilian population in its African origins.^1^ The analyses were performed by geneticist Sérgio Pena of the Federal University of Minas Gerais, who has conducted extensive research on the ancestry of the Brazilian population. Neguinho’s test revealed 67.1 percent European ancestry.^2^ Pena considers this typical of the high levels of genetic mixture among Brazilians that his studies have systematically revealed since the publication of his ‘Molecular Portrait of Brazil’ (Pena et al., 2000).

The results of the project were reported in national mass media, and Neguinho’s case figured in heated debates about the affirmative action policies aimed at Brazil’s black population, such as quotas for access to public universities and differential health policies. A long-standing goal of the black movement, supported by a number of social scientists, such policies were implemented by the Brazilian state in the mid-1990s with the objective of redressing social inequalities that in Brazil are strongly correlated with skin colour (Htun, 2004). Race-based affirmative action, however, met strong resistance from various sectors – including centre-right and far-left political parties, the mass media and some social scientists – who considered such policies inappropriate for a country as racially mixed as Brazil, and proposed class-based criteria for inclusion instead (Fry et al., 2007). Genetic data indicating the ‘biological nonexistence of race’ and that ‘all Brazilians are *mestiços*’ were used in arguments against race-based affirmative action policies, which became particularly noticeable in the media hype around Neguinho’s case. Lawyer Roberta Kaufmann, author of a petition filed with the Brazilian Supreme Court to have racial quotas declared unconstitutional, alluding to the argument that reparations for slavery justified such policies, argued that

It is more probable that Neguinho da Beija-Flor is a descendent of a person who owned slaves than of a black person who was enslaved … For this reason, there is no way you can justify racial quotas. (Kaufmann, 2011)

In addition, Neguinho’s case was used to deconstruct the very idea of a black identity. Kaufmann affirmed that ‘he should have been called Branquinho [Whitey] da Beija-Flor.’^3^ In contrast, Neguinho dismissed the results as irrelevant for defining his identity: ‘Me, European? A black guy like me! I’m going by skin colour. If I said that I’m 67% European, people would think I’m messing around with them.’^4^ Black activist Frei David, who also had been tested as part of the Afro-Brazilian Roots project, affirmed,

I’ve never seen any police raid, in a bus for example, where they asked people what percentage of Afro genes they had before discriminating against them … The discriminator does not see in genetics any argument to stop discriminating. However, they want the discriminated to stop fighting for their rights because ‘we all have Afro genes’.^5^

David dismissed the relevance of genetics for racial classification and racism in Brazil, and questioned the political motivations behind the use of genetic data, suggesting that it undermined the black population’s struggle for racial equality.

The incorporation of genetic data and arguments into the debate on race and affirmative action in Brazil offers a valuable case to explore questions about the relationship between genetics, politics and social identity. How does a political use of genetics against race – as proposed by some geneticists and social scientists – play out in practice? What purchase do genetically inflected interventions have in the political domain? And how do affected groups such as the black movement respond to the use of genetic data – a question that has been little explored for Brazil (but see Santos and Maio, 2005)? The case highlights the influence of political processes and unequal power relations in shaping the outcomes of this relationship in everyday practice, and into different ways in which genetics figure in political arguments for and against race-based affirmative action policies in Brazil – publicized in dramatic fashion by Neguinho’s case.

Data collection for this article was conducted by Michael Kent in 2011 and 2012 during fieldwork in Brazil, mostly in towns with a strong presence of black movements,^6^ including São Paulo, Porto Alegre and Rio de Janeiro. A wide range of visual and written material was collected, including blogs, newspaper articles, postings at online discussion forums, policy document and juridical documentation, and videos of public debates, meetings and juridical hearings. Focus groups were held with students in social and medical sciences at universities in Rio de Janeiro and Porto Alegre. Kent attended public debates on affirmative action, as well as meetings of the black and pro-quota movement. He interviewed approximately 30 members of the black movement and other pro-quota activists individually, while some 50 more participated in eight collective conversations. Black movement activists frequently requested that exchanges should be of mutual benefit, with the sharing of information and knowledge going both ways. As a result, a series of workshops or collective conversations were held, starting with discussions on participants’ perspectives on and experiences with the debates about race and genetics, followed by Kent’s presentation on technical aspects of genetic ancestry research, its relation to notions of race and identity, and studies on the Brazilian population conducted by Pena and others. In such meetings, data were collected before the presentation in order to avoid biasing results. Earlier, Kent conducted laboratory ethnographies in several of Brazil’s main centres of genetic ancestry research in order to capture their perspective on the relation between genetics, race and identity. Before presenting the results of this research below, we first discuss the relation between genetics and racialized identities, outline historical debates on race in Brazil and describe Pena’s research on the genetic ancestry of the Brazilian population.

## Genetics, race and identity

One strand of science studies focuses on the persistence and even re-emergence of biologized versions of race or racialized constructs within recent genetic research itself, despite declarations that genetics provides definitive proof about the nonexistence of biological race. This may be because a minority of geneticists think that race is a useful category, biologically speaking (Burchard et al., 2003), or because they see it as an important category in a struggle for social inclusiveness in science and medicine (Bliss, 2012). Or it may be because racialized constructs persistently re-appear in biological science, often against the grain of the scientists’ intentions but woven into their standard practices and assumptions about populations as simultaneously social and biological entities; this creates a constant slippage between the two aspects, thus potentially underwriting social categories with an explanatory, predictive biology.^7^

A second, overlapping, strand in science studies focuses on how genetic knowledge interacts with domains of knowledge beyond the research laboratories. One question concerns whether or not widely circulating concepts of racial and other social identities (ethnic groups, nations) become geneticized – bearing in mind the diverse possibilities that genetic science offers for relating the social and biological dimensions of human groups. This entails a broader question about how different forms of knowledge come to have authority to make claims about matters of fact and matters of value in the public domain. Research in this strand indicates the indeterminacy of genetic knowledge, despite the common image of genetics as a ‘truth machine’ (Lynch et al., 2008). DNA ancestry testing, for example, is a field of contested knowledge, where the limits of scientific knowledge are debated (Bolnick, 2008; Bolnick et al., 2007) and where the users of that knowledge deploy genetic data in inventive, hybrid and selective ways, crafting narratives about origins, belonging and entitlement in ways that cohere with their projects, individual or collective.^8^

DNA data do not determine social action, but instead provide diverse possibilities, interacting with other knowledges. Ideas about the relationship between national identity and race influence how DNA databanks are organized in Canada and Iceland, for example, and how a national ancestry-testing project is constructed in Britain (Hinterberger, 2012; Nash, 2013; Pálsson and Rabinow, 1999). Existing social categories, such as the Brazilian categories of black or brown, have circulated for centuries through scientific, bureaucratic, political and everyday domains (Santos et al., 2014). These concepts now act as organizing categories for genomic science, as they did for earlier genetic studies (De Souza and Santos, 2014). In the process, they gain new genomic meanings (as they did for Neguinho). For some people, to what ‘blacks’ are entitled in Brazil is shaped by whether ‘blacks’ can be said to exist as a biological category – a question that others deem irrelevant to issues of entitlement. In this sense, the category ‘black’ is co-produced as it circulates through genetic and political domains (Jasanoff, 2004; Reardon, 2008).

In the United States, racial categories are institutionalized by clinical practices, medical research and government bureaucracies as part of a broader movement towards social inclusion positing that people must be counted by race to prevent racial exclusion (Epstein, 2007). In contrast to the United States, in the Brazilian social inclusion movement, which also seeks to correct racial exclusion and also counts by race, genetic data are interpreted as showing generalized mixture, rather than discrete racial groups, so little fear is expressed that race will become geneticized. This is despite the fact that the genetic emphasis on mixture depends, as such ideas always do, on the assumption that racial groups existed somewhere or sometime else.

The Brazilian example contributes to existing debates in science studies by showing how genetic knowledge, rather than transforming ways of conceiving social identity and belonging, circulates in ways powerfully shaped by the existing socio-racial order even as it simultaneously provides new tools for thinking about that order. The Brazilian case shows the paradoxical routes genetic knowledge can take, due to its ambivalent potential and uneven traction. It illustrates that contradictory processes of de- and re-racialization, and of re- and de-geneticization can take place simultaneously, as debates rage back and forth in Brazil about the appropriateness of marking racial difference in a racially mixed society. It shows how issues of citizenship can purposely marginalize the relevance of genetic knowledge in some spheres, despite its authoritative status.

## Race, mixture and nation in Brazil

In the 19th and early 20th centuries, scientific interpretations of race and the biological constitution of the country’s population played an important role in debates about the nation. In scientific racist and eugenic theories, white and black Brazilians constituted distinct racial types – with a marked superiority of the former – and inter-racial mixing was thought to result in the degeneration of the Brazilian population, compromising its future viability as a nation. This spurred policies to whiten the population by encouraging the immigration of approximately six million Europeans (Santos et al., 2014; Schwartz, 1993; Skidmore, 1993; Stepan, 1991).

Since the 1930s, race mixture – commonly referred to as *mestiçagem* – often has been reinterpreted in positive terms and the hybrid figure of the mestiço became central to the construction of a unified national identity. Cultural interpretations of the Brazilian population displaced biological approaches in public debates. The work of Gilberto Freyre has been particularly influential, with his argument that the intense and relatively consensual mixing of white Europeans, black slaves and Indians blurred differences to such a degree that at present there are no clear-cut distinctions between them, only a racial continuum from the whitest to the blackest individual (Freyre, 1936). From here, it has often been argued that the absence of clear divisions has allowed Brazil to become a ‘racial democracy’ in which relations between people of different colours are relatively egalitarian and racism plays a minor role. The elevation of racial democracy to the status of national ideology from the 1930s onwards, however, did not supplant racialized social hierarchies nor the ideal of whiteness, which continued to figure prominently alongside celebrations of mixture (Twine, 1998).

Freyre’s (1936) view of Brazilian society has been challenged since the 1950s – and especially since the late 1970s – by social scientists and black activists who highlight the continued existence of profound racial inequalities. The concept of racial democracy is now recast as a myth that perpetuated racism by denying its existence. The alternative interpretation that Brazilian society consists of economically and socially differentiated white and black (or non-white) segments has become increasingly articulated (Guimarães, 1999; Hanchard, 1994; Hasenbalg, 1979; Hasenbalg and Silva, 1992; Telles, 2004). From the mid-1990s, this prompted the adoption of public policies aimed at Brazil’s black population, including affirmative action measures such as racial quotas in public employment, specific health policies and the recognition of land rights of *quilombo* (maroon) communities (Htun, 2004). Quotas for access to public universities – which in Brazil are of higher quality than private ones – have become a particular focus, justified on the grounds that in such universities there is a disproportionate enrolment of white students, up to 96 percent in elite courses such as medicine (De Carvalho, 2005: 36).

Affirmative action policies are debated in heated terms, supported by parts of the political and academic establishment and the black movement, while challenged by segments of the national intellectual elite, the mass media and – mostly centre-right, but also some left-wing – political parties, who have proposed race-blind socio-economic criteria for inclusion instead. Their critique centres on arguments that in Brazil: (a) inequalities are more a question of class than race, (b) it is impractical to define who is ‘black’ in a country with such a mixed population and (c) race-based public policies might prove counter-productive by reinforcing the categories on which racism is based (Fry, 2005a; Fry et al., 2007; Magnoli, 2009; Maio and Santos, 2005).

In this context, taxonomies based on race, colour and descent co-exist, rather than being mutually exclusive. The national census includes the main colour categories of *branco* (white), *pardo* (brown) and *preto* (black). In contrast, the black movement and some social scientists propose a racialized binary system of *branco* and *negro* (including pardos and pretos). Everyday folk taxonomies include a myriad of intermediary categories. While the predominant criterion for classification is an individual’s appearance, cultural repertoires, class distinctions and perceptions of European or African origins also play a significant role in identity constructions. Overall, a permanent tension exists between a bi-polar principle of classification and a more flexible and continuous modality (Fry, 2005a; Telles, 2004; Twine, 1998). This contested taxonomic and political field is the context for Sérgio Pena’s genetic research.

## Sérgio Pena, race and the genetic ancestry of Brazilians

Coming from a background in medical and forensic genetics, Sérgio Pena became interested in the ancestry and diversity of indigenous and other populations in Brazil. From 2000, Pena and colleagues at the Universidade Federal de Minas Gerais published studies with samples from various regions of Brazil, analysing mtDNA, Y-chromosome and autosomal DNA markers (for overviews, see Pena et al., 2009, 2011). Pena has emerged as a public scientist and intellectual who, in popular as well as academic publications, addresses issues of social policy and national identity from the perspective of genetics. He emphasizes the high levels of genetic diversity in Brazil and sees this as good evidence for the nonexistence of race as a biological reality. He argues that this information is relevant for medicine and policy-makers in Brazil (Kent et al., 2014; Santos and Maio, 2004; Wade et al., 2014). Pena’s work is also discussed in Kent et al. (2015), in this special issue.

Pena’s research on the autosomal DNA of individuals identified as white, *pardo* (brown) and *preto* (black) revealed much overlap in genetic ancestry between the three categories; there was a weak correlation between people’s skin colour and their genetic ancestry (Parra et al., 2003). Pena has since analysed the mixed genetic ancestries of a variety of Brazilian samples. A recurring element is an emphasis on the high levels of non-African ancestry found among individuals identified as brown or black. Pena’s most recent work concludes that among populations across Brazil European ancestry is predominant, even among ‘non-white’ – that is, black and brown – individuals, a result that Pena attributes to European immigration (Pena et al., 2011).^9^

Pena’s research has been disseminated widely. He has published in social science journals in Brazil, mostly as part of special issues focusing on race and affirmative action (Pena, 2005; Pena and Birchal, 2006; Pena and Bortolini, 2004). He also has written newspaper columns, made frequent media appearances and published popular scientific books (Pena, 2008, 2009). His work bridges genetics and debates on race and Brazilian national identity. Pena consistently argues against the use of the concept of race both in medical research and in general, and actively engages in the debate on racial quotas and differential health policies targeted at the black populations (Pena, 2005; Pena and Bortolini, 2004). He acknowledges that in spite of its nonexistence at the genetic level, race does exist as a social construct. However, making reference to cultural theorists such as Paul Gilroy, he defines social race as toxic and has made it his mission to ‘un-invent’ race and contribute to the creation of a de-racialized society (Pena, 2008, 2009; Pena and Birchal, 2006). In the debates on university admission, he was involved in the political campaign against racial quotas, giving evidence on the nonexistence of race at the genetic level in the 2010 Supreme Court hearings on the constitutionality of such quotas. But he represents both science and his own role in these debates as being apolitical: ‘modern genetics can offer support for political decisions and … the genetic profile of the Brazilian population should certainly be taken into account in political decisions. But genetics cannot claim an explicitly prescriptive role’ (Pena and Bortolini, 2004: 46).

Initial public reactions to Pena’s research were indicative of the debate that developed in following years. Political commentator Elio Gaspari (2000) hailed the research as ‘scientific proof of what Gilberto Freyre formulated in sociological terms’. In the same year, black anthropologist Athayde Motta warned in *Afirma: Revista Negra Online* that the results might be used for ‘a pro-racial democracy campaign … to maintain the state of racial inequality in Brazil’ (cited by Santos and Maio, 2004: 351). Indeed, genetic data became a recurring element of arguments against affirmative action.

## First clashes: Genetics, whiteness and African ancestry

Genetic arguments entered the affirmative action debate when the first quota system was established in 2003 at the public University of the State of Rio de Janeiro (UERJ), which reserved 40 percent of all places to applicants who declared themselves ‘negro’ or ‘pardo’. Although the measure triggered strong opposition – even within the university – elite private schools and university preparatory courses reportedly encouraged students to apply for a racial quota at the UERJ – irrespective of their own race or skin colour – on the grounds that as Brazilians they were likely to have black ancestors. Interviewees reported that in 2003 some quota positions were filled by white students who had self-identified as ‘pardo’, often justifying their choice with reference to a black (great)grandparent. When black activist Frei David visited the UERJ to question this practice, he reported receiving the following reply from the Rector, who at the time opposed the quotas: ‘Look Frei, who is going to prove that these people are white? They have a white skin, but genetically they are Afro…. The law is open, they self-declared like this, and that’s it.’^10^ In following years, some universities tried to vet applicants, using photographs and interviews (De Carvalho, 2005; Maio and Santos, 2005), leading some applicants to have their claims rejected on the grounds of their appearance. In an interview, Pena reported that a number of these contested the decision in court, using evidence of African genetic ancestry provided by his DNA tests (Gomes, 2007).

Genetics became an element in personal racial classification and the proverbial ‘black great-grandmother’ originally deployed in the quota debate was given new genomic meaning and rearticulated as a percentage of African genetic ancestry. Genomic science mobilized the notion of an invisible, impersonal genetic ancestry in the individual body, making it feasible to claim to be brown or black – in the context of a racial quota application – while still appearing white and enjoying the everyday privileges thereof. This blurred the boundaries between the established racial categories used in such policies, and redefined all Brazilians as potential beneficiaries of quotas reserved for *negros*. Claims to entitlement were being defined as applicable to all Brazilians, united in their mixed genetic ancestries, contesting the idea that a specific racial category could exist as a subject of entitlement.

## The political use of genetics against affirmative action

As more Brazilian public universities adopted affirmative action policies from 2004 onwards, and as the political debate became ever more heated, references to genetics have become increasingly prominent in arguments against such policies. A number of social scientists support race-based affirmative action^11^ and, in doing so, a few of them address genetics (Dos Anjos, 2005; Munanga, 2004, 2005). More evident is the way several social scientists draw on aspects of Pena’s research to criticize race-based policies, even if some of them diverge from Pena’s thoroughgoing advocacy of colour-blindness in all aspects of social policy. They use his data in academic publications (Fry, 2005a: 15, 297–298; Magnoli, 2009; Maio and Santos, 2005: 203–205), including in co-publications with Pena resulting from collaborative research (Pena and Birchal, 2006; Santos et al., 2009), in interventions in the media (see examples in Fry et al., 2007) and in what came to be known as the anti-quota manifesto (Daher et al., 2008).

The mass media – in particular the *Globo* TV and press network, *Folha de São Paulo* newspaper and *Veja* magazine – play a prominent role in the dissemination of Pena’s research and genetic-based arguments against affirmative action.^12^ The Afro-Brazilian Roots project became a potent vehicle for this, as the ancestry of Neguinho da Beija-Flor gained an iconic quality that appealed to popular imagination. With few exceptions, it is through media coverage of Neguinho’s ancestry that black activist interviewees first confronted genetic arguments against affirmative action. Finally, genetics features prominently in the petition that the Democratas political party presented to the Supreme Court to have racial quotas declared unconstitutional, and which includes an expert opinion by Pena (Kaufmann, 2009: 27–37, 132–179). In such uses, genetics is systematically presented as neutral, objective knowledge.

Genetic data and arguments are deployed in the affirmative action debate in three main ways: to deny the existence of human races in general; to deny their relevance specifically for Brazil and to deconstruct black identity. Turning to the first use of genetics, the anti-quota manifesto, for example, stated that ‘human races do not exist. Genetics has proven that the iconic differences of the so-called human “races” are superficial physical characteristics’ (Daher et al., 2008). As Globo Network’s director of journalism Ali Kamel (2006) claimed in his anti-quota book *Não somos racistas* (we are not racists), the consensus among geneticists is that ‘all humans are equal’ (p. 51).

Second, the high levels of genetic mixture revealed by Pena’s research are deployed to affirm the particular salience of such points for the Brazilian population: ‘We [Brazilians] are, thank God, a total mixture’ (Kamel, 2006: 55). The lack of correlation between genetic ancestry and physical appearance among the Brazilian population emphasized by Pena’s research (Parra et al., 2003) is used to deconstruct phenotypic boundaries. The petition to the Supreme Court referred to above uses such evidence to question the possibility of defining who is black in Brazil and who qualifies for racial quotas. The petition concludes that ‘if it is not possible to define objectively, without a margin of doubt, the true beneficiaries of a public policy, then its efficacy will be null’ (Kaufmann, 2009: 36). Interestingly, while genetics is often associated with the establishment of certainty – for example through paternity tests and the forensic identification of bodies – in this case, it has principally been used in order to cast doubt. This approach shifted the focus away from the social inequalities emphasized by the black movement and proponents of racial quotas towards the issue of classification and the (non)existence of difference.

Such uses of genetics suggested a primacy of biological over social definitions of race. The authors of the anti-quota manifesto, for example, quoted Pena to affirm that: ‘the scientifically proven fact of the nonexistence of the “races” must be absorbed by society and incorporated into its convictions and moral attitudes. A coherent and desirable attitude would be to construct a de-racialized society’ (Daher et al., 2008). The biological nonexistence of race was used to invalidate race as a social construct and genetics was seen as a mandate for the social order.

The research conducted for this article sheds light on these complex circumstances. The experiences of the black activist interviewees illustrate the ways in which the genetic nonexistence of race – in Brazil or elsewhere – is used against affirmative action. Many interviewees said that they are confronted in public debates with genetic arguments as formulaic anecdotes, rather than through discussions of scientific detail. The most recurrent were ‘according to genetics we are all equal’; ‘genetics has proven that race does not exist, therefore it is impossible to have racial quotas’ and ‘if even Neguinho da Beija-Flor is 67% European, it is impossible to define who is black in Brazil.’ Such references to genetics occurred mostly in articulation with pre-existing arguments against affirmative action that focuses on the pervasive mixture of the Brazilian population, the idea of racial democracy or a national identity that transcends racial differentiation. Reflecting on his experiences, economist Marcelo Paixão argued that ‘genetics seemed almost like a measure of despair, to revive something [racial democracy] that was already dying.’ As such, genetic data were part of broader arguments, which they reinforced with scientific authority.

In the wake of the Afro-Brazilian Roots project, Frei David had the relevance of his political battle repeatedly questioned during interviews with national media. According to David, he was asked ‘whether given the project’s results it made any sense to continue fighting, whether working on the issue of the black population would not bring a disharmony to Brazil that does not exist.’ And Edson França, national coordinator of Unegro – the black movement affiliated with the Brazilian Communist Party – showed me a dossier with the party’s ‘strategic readings’, which included Pena’s article *‘*Molecular Portrait of Brazil’. As França explained,

This article was used in order to discuss the issue of the Brazilian population. In general, people are delighted with it. It’s a very comfortable discourse – it’s like saying ‘there aren’t any black people, there is no race, so we don’t have to talk about this.’ … The only ones who contest this are people related to the black or indigenous population.

Genetic data had become institutionalized as part of the affirmation of the primacy of class over race.

A third way in which genetic arguments are used in the affirmative action debate is in the deconstruction of black identity, in particular through references to Neguinho da Beija-Flor’s European ancestry. Several black activists recalled how they were mocked in the wake of the Brazilian Roots project, including Ana Honorato of the Movimento Negro Unificado: ‘people started telling us “you’re not black, you’re just a bunch of white guys [*um bando de brancos*].”’ While this seems to have had little effect on people who strongly identified as black, some interlocutors mentioned public figures or acquaintances who started downplaying their own blackness using genetic arguments, apparently seeking to avoid the stigma attached to being black. And after the media coverage of Neguinho’s ancestry, several schools in Rio de Janeiro reportedly encouraged students to search in their family history for European ancestors, as part of class projects. Genetics became a means to prioritize and accentuate the value of Brazil’s European heritage.

The *Globo* newspaper published a front-page feature about Pena’s recent research (Pena et al., 2011) with the title ‘a more European country’, redefining Brazilians as ‘Brazipeans’ (‘*brasipeus*’ – a combination of *brasileiros* and *europeus*) (Globo, 2011b). The editorial affirmed: ‘science has proven the nonexistence of the Afro-Brazilian’ (Globo, 2011a). The use of genetics against affirmative action began to affirm the irrelevance of such policies by downplaying the size of the black or Afro-descendent segment of the population. As European origins are closely related to whiteness in Brazil, such uses of genetics turned it into an additional avenue for strategies of whitening.

In spite of genetics’ role in political strategies aimed at the deconstruction of the idea of race, genetics has also been used to reinforce pre-existing understandings of race. Media features of Pena’s research frequently employ terms such as ‘black genes’ or ‘white ancestry’, while Neguinho and black activists are redefined in racial terms as being ‘white’ on the grounds of European ancestry. And one of the questions raised by the petition to the Supreme Court was, ‘What if a person is black in his ancestry, but white in his appearance?’ (Kaufmann, 2009: 31). This substitutes Pena’s conceptual separation between genetic ancestry and the phenotypic markers of racial identity with a conflation of the two. Genetics is used in order to speak precisely about race in biological terms, rather than to undermine its existence. The ways in which genetic data and arguments have been appropriated in the political debate often resonate closely with popular understandings of race, origins and appearance and not with geneticists’ own approach to race.

## The black movement’s disengagement from genetics

How, then, have members of the black movement dealt with these genetic arguments? When the Afro-Brazilian Roots project and Neguinho’s European ancestry were disseminated by the media, genetics was for a time intensely debated among Brazil’s black movements and their networks, during meetings, in mailing lists and the social media.^13^ Two broad positions emerged: while some advocated challenging the genetic argument, others preferred to ignore it or affirm its irrelevance to the debate. The latter approach eventually prevailed, for three main reasons.

First, many members of the black movement chose not to challenge the content of genetic arguments because they welcomed the notion that all humans are biologically equal, given the historical antecedents of racial discrimination justified by black people’s supposed biological inferiority. Second, those in favour of challenging the genetic arguments did not find the means to do so effectively. Engaging with genetics was made more difficult by what Stenio Rodrigues – director of the Ministry of Health’s audit unit in Porto Alegre – called in a research interview ‘the deification of scientific knowledge as absolute truth’. As such, it required ‘following the same rituals of academia’. In this vein, Humberto Adami – currently ombudsman of the federal government’s Secretariat for Racial Equality – at the time argued in a Yahoo discussion group on racial discrimination for the need to develop counter-arguments from within the same scientific field:

[this] research appears to be one more of those that will be quickly unmasked … The adequate way forward would be to search for similar research in other places, or people from the same field, that will confront it ‘genetically’.^14^

As Rose Torquato – Secretariat of Racial Equality of São João de Meriti in the Rio de Janeiro metropolitan area – explained in a research interview, ‘this is the question: you need to have knowledge; it’s about knowing what weapons are being used in the debate in order to defend yourself on an equal footing.’

In fact, several prominent actors in the black movement searched for black or pro-quota geneticists who could help them in formulating a scientific counter-argument. These included Frei David, members of the Movimento Negro Unificado (Unified Black Movement) and the Affirmative Action work group of the Universidade Federal do Rio Grande do Sul. However, they found none. While Frei David secured the assistance of Rosa Andrade, a black geneticist specialized in sickle-cell anaemia, she considered the technical knowledge involved in ancestry research an obstacle to challenging its content. In addition, the substantial resources required for such research made it prohibitively expensive (cf. Latour, 1987). As it proved impossible to mobilize scientific experts and their knowledge for the production of a counter-argument, or even to gain sufficient working knowledge of genetics to engage the scientific debate, members of the black movement instead focused on keeping genetics outside the affirmative action debate. As such, genetic knowledge was deployed mostly by opponents of affirmative action. This led several interlocutors to conceptualize genetics in Brazil as a racialized scientific field, illustrating the necessity for the quota system, as well as the political cost of their under-representation in higher education and scientific knowledge production.

Third, most interlocutors in the black activist movement interpreted the use of genetics by their opponents as a strategy to de-politicize the debate on affirmative action. It shifted the focus away from structural social *inequality* between black and white segments of the Brazilian population towards the nonexistence of biological *difference* and the difficulty of identifying the beneficiaries of racial quotas. As Antonio Matos – the Movimento Negro Unificado – explained, ‘contesting the genetic argument would mean accepting its legitimacy and place in the debate; we had to get back to the social aspects of the debate.’ Ignoring the issue of genetics altogether was a strategy to keep the focus on the social dimensions of race.

This disengagement from genetics represented a departure for the black movement – and others campaigning in favour of racial quotas – which had developed extensive counter-arguments to all other objections raised against racial quotas. For example, the 2010 Supreme Court hearings on the constitutionality of quotas pitted historians against historians, sociologists against sociologists, one set of statistical data against another, and legal specialists arguing over Articles 3 and 5 of the Constitution. In contrast, Pena was the only geneticist who participated in the hearings.

However, given the incorporation of genetic data into anti-quota arguments, merely ignoring the issue was not sufficient. Genetic arguments had to be actively kept outside of the debate, by affirming their irrelevance. Two interrelated arguments became key to this: that in Brazil race functions as a social – rather than a biological – category, and that racial classification and discrimination in this country are based on appearance, rather than on genotype. Such responses initially developed within localized contexts and became more standardized through exchanges within the black movement and through the 2008 pro-quota manifesto, which included an extended refutation of the relevance of genetic arguments (Do Nascimento et al., 2008: 17–19).

## Affirming the irrelevance of genetics: Social race versus biological race

Separating social from biological dimensions of race – and placing the debate on affirmative action squarely within the social domain – was a central element in the strategy aimed at keeping genetics outside of this debate. In response to the widespread accusation that the black movement and affirmative action policies were resurrecting a biological notion of race, the pro-quota manifesto of 2008 stated, ‘why do they insist on denying something that no one affirms? … We defenders of quotas never talk of race in the biological sense of the term’ (Do Nascimento et al., 2008). For black geneticist Rosa Andrade, the concept of race had very different meanings in biology and in the social sciences, and the projection of a biological definition of race onto discussions about racism and social inequality was a constant source of misunderstanding in the affirmative action debate. As she commented in a research interview: ‘this kind of knowledge [genetics] needs to stay within its correct place.’

Several elements were adduced in seeing race as a social phenomenon. Statistical data revealing the existence of structural inequalities in income, education and health have played a central role. As Angelica de Jesus Santos – Secretary of Racial Equality of São João de Meriti – explained, ‘what we could do was to show a multitude of socio-economic indicators that show that being black in Brazil is different from being white.’ This draws on a well-established sociological tradition of using quantitative data to systematically reveal the structural character of racial inequalities in Brazil (Hasenbalg, 1979; Hasenbalg and Silva, 1992). Economist Marcelo Paixão used this strategy pre-emptively to avoid discussing genetics:

I direct public debates immediately towards socio-economic indicators. When I start my talks, I ask ‘does race exist? Genetics says this and that … But socio-economic indicators show …’ Because I don’t want genetics to get into the debate.

This established a conceptual separation between a genetic dimension of race – in which all can be equal – and a social dimension in which structural inequalities persist. This persistence is invoked as another argument for the irrelevance of genetics to the debate. According to Wilson Prudente, public prosecutor in Rio de Janeiro, ‘genetics has been saying for the last ten years that we are all equal, but this hasn’t changed anything in the levels of racism in Brazil.’

This distinction between the social and biological dimensions of race was deployed in order to argue for the primacy of social science over genetics in dealing with race. As Stenio Rodrigues observed, ‘as much as they try with genetics to deconstruct [race] – there is a reality that exists independently of scientific proof…. Genetics does not explain social inequalities.’ And during the preliminary speeches to the Supreme Court’s decision on the constitutionality of the quota system in 2012, public prosecutor Indira Quaresma argued that

We can’t afford to think that the natural sciences have primacy over the social sciences …. Racism is a fact of life in society, an absolute problem of the social sciences, and it is in [these sciences] that its end must be looked for.

In addition, because genetic arguments were often used in order to deny the possibility of defining who is black, public debates – as well as the conversations conducted during research – often emphasized a wide range of social elements thought to define blackness. Such markers of a distinctive black culture included forms of music, dance, food, the *capoeira* martial art, participation in religions of African origin such as *candomblé*, and a shared history of slavery and discrimination. Descent also played a role, as some interlocutors highlighted origins in the African continent, and identified as African, Afro-descendent or Afro-Brazilian. Descent, however, was mostly framed in spiritual, religious and cultural terms, rather than in a biological language. Physical appearance, and in particular skin colour, was also a key factor. Self-identification played a central, but far from exclusive role. For many, being identified as black by others – mostly on the basis of appearance – and suffering everyday experiences of discrimination as a result was just as important in acquiring a racial consciousness: ‘no one is born black in Brazil, you become black’ was a frequent comment.

This importance of appearance in the construction of blackness in Brazil – both in terms of self-identification and hetero-classification – provided a second key element for the black movement’s response to genetic arguments: racism in Brazil is based on appearance, rather than on origin or genotype. This was important to emphasize, as arguments based on ancestry had been used to justify ‘white’ students applying for racial quotas, as noted earlier. A recurrent trope of counter-arguments is that if you want to know who is black in Brazil, just ask a policeman: he does not need a genetic test before identifying who is black. And when Indira Quaresma participated in public hearings at the Legislative Assembly of the state of Rio Grande do Sul on the adoption of racial quotas in public sector employment, she dismissed the relevance of genetics as follows:

They argue … that Neguinho da Beija-Flor has more than 60% of European DNA, and therefore should be called Branquinho da Beija-Flor. … Yes, we are mixed. But racism in Brazil, as Oracy Nogueira said, is based on appearance, and not on origin. We Brazilians observe phenotypes and not genotypes. This is because families whose members have different colours are much more common than people think. Mine, for example. I’m the daughter of a black woman and an Indian man. My parents divorced, my mother remarried and my half-sister is as white as can be. My white sister is as Afro-descendent as I am, but she has never suffered, nor will she ever suffer, situations of prejudice, discrimination or racism because of this.

Nogueira’s (2006 [1954]) distinction between racial classification based on appearance in Brazil and on origins in the United States was also used by defenders of affirmative action when the adoption of a quota system was debated at the Universidade Federal do Rio Grande do Sul in 2007. The biology department organized well-attended public seminars in which genetic arguments against racial quotas figured prominently. The University’s pro-quota Affirmative Action workgroup – consisting of academics, students and black activists – parried such arguments by drawing on Nogueira. According to Luanda Sito, one of the workgroup’s founding members, ‘this was the key to avoid having to deal with genetics – if racism in Brazil is based on appearance, and genetics speaks about origins, then genetics has no relevance for the debate.’ In other words, even if it is not possible to define who is black in Brazil at the genetic level, it is certainly possible to make such definitions at the level of appearance, and such definitions are in fact routinely made as part of everyday social life.

This distinction became entangled with parallel divisions between the inside and the outside of the body and between what is visible and invisible. As geneticist Rosa Andrade, for example, observed: ‘genetics says that you can’t have quotas because we’re all equal. OK, but we’re only equal up to the walls of my cells.’ Altiva, São João de Meriti’s Secretariat of Human Rights, argued in a similar vein that ‘you can turn me inside out and show that I’m European on the inside, but to others I will continue to be black.’ The ways in which opponents of affirmative action draw on genetic research to disqualify phenotypic identifications as ambiguous has resulted among black activists in an understanding of genetics that equated it with ancestry and genotype. Yet, phenotypic characteristics such as skin colour have a genetic basis too. When this issue was raised during conversations, interlocutors sometimes responded by establishing an additional separation between the ‘visible side of genetics’ and its ‘invisible’ side.

As such, the conceptual separation established between the social and biological dimensions of race is understood to run through individual bodies as well. On the one hand, there is a social domain of the body, associated with skin colour, phenotype, the visible exterior, being black or white, and which concentrates the social gaze and the attachment of meanings. On the other hand, there is a biological domain of the body, associated with genotype, the invisible interior, being of African or European ancestry, and which is not so easily available for social signification.

Such arguments about the irrelevance of genetics for the affirmative action debate based on the distinction between social and biological dimensions of race were brought to the Supreme Court during its public hearings on quotas. Several speakers on the pro-quota side made brief references to genetics in order to dismiss its relevance. Although some interlocutors worried that neutralizing genetic arguments – rather than refuting them – would contribute to their pervasiveness, the strategy eventually proved successful. In 2012, the judges of the Supreme Court unanimously voted in favour of the constitutionality of the quota system. In his verdict, the judge responsible for reporting on the case – Ricardo Lewandowski (2012) – said this:

Although today from a scientific perspective no subdivision of the human race is recognized anymore, racism persists as a social phenomenon, which means that the existence of the diverse races results from their mere historic, political and social conception, and it is this conception that must be considered in the application of Law. (p. 19)

Lewandowksi (2012) concluded that ‘it is necessary, for the purpose of this discussion, to remove the biological concept of race’ (p. 20). Following the Supreme Court’s decision, Brazil’s federal government passed a law in 2012 making the establishment of quota systems based on both racial and socio-economic criteria compulsory for all public universities.

## Discussion: Genetics, politics and race

Brazil’s affirmative action debate offers a compelling context in which to explore how the politicization of genetics as a weapon against race plays out in practice, and raises wider questions about the relationship between genetics, politics and identities. The incorporation of genetic data into this debate works with a well-established conceptual separation between nature and society. Opponents of affirmative action emphasize the lack of correlation between genetic ancestry and racial identity in Brazil as part of their efforts to delegitimize the concept of race on which such policies are based. They want nature to act as a mandate for society: racial categories do not exist genetically; therefore, they cannot form the basis of rational social policy. These critics often use the conceptual separation to question the relevance of race as a social construct, thereby establishing – or at least implicitly assuming – a primacy of biological over social definitions of race. In contrast, members of the black movement employ the same nature/society distinction in order to claim that genetics is irrelevant for a debate centring on socially produced identities and inequalities, thereby re-affirming the primacy of social conceptions.

This Brazilian material indicates the very uneven ways in which genetic knowledge flows through social domains, indicating the complexity of the way processes of geneticization and molecularization work in relation to society and race (Heath et al., 2004; Rose, 2007; Rose and Novas, 2005; Schramm et al., 2012) and indeed showing how contrary effects can occur simultaneously. Those in favour of race-based affirmative action are re-racializing Brazilian society by arguing for the social relevance of racial identities and differences as a basis for public policy. At the same time, in arguing for the irrelevance of genetics to the debate on affirmative action, they are de-geneticizing both race and society. For black activists, physical appearance is a key criterion for defining race, and thus phenotype is held apart from genotype. Phenotype is taken as a social fact: ask a policeman who is black, not a geneticist. Although genetics figures in debates about citizenship here – in the negative sense of being considered irrelevant – this is the very opposite of the idea of an increasingly geneticized mode of citizenship in which new forms of sociality are based on ideas of genetic connection and belonging.

Meanwhile, critics of quotas fear the re-racialization of Brazilian society, but the possibility that biological knowledge was genetically reifying race in Brazil does not concern them, because the genetic data are taken to show generalized mixture – understood in positive terms – rather than differentiated racial categories. In other contexts, some fear that the geneticization of race may work against racialized minorities, by biologically reifying them and creating tools for their exclusion (Duster, 2006); however, in Brazil some worry that genetic accounts of race, rather than reifying the black category, would dissolve it and thus undermine black political solidarity (cf. Kent, 2013). The effects of geneticization of identities and public debates are highly uneven. Those who deployed genetic arguments against racial quotas and even against the existence of a black category in Brazil – and we must recognize the different ways in which mass media, social scientists and geneticists used genetic data – at one level attempted to de-racialize Brazilian social relations (as a sub-set of human relations), but simultaneously to re-geneticize society.

Paradoxically, the same genomic arguments about the nonexistence of biologically differentiated racial categories can work in a different way to re-racialize Brazilian society. The genetic data are used to argue that everyone is more or less mixed and that Brazil is a heterogeneously *mestiço* nation. But this argument easily resonates, in the wider public sphere, with a racialized conception of the nation, understood as a population that has been constituted by the mixture of three original racial stocks: the concept of ‘*mestiço*’ has great difficulty in shaking off its racialized genealogy (Young, 1995), even if the geneticists argue that race has no biological reality in general and specifically in Brazil. Thus, the re-geneticization of the social order makes more available new types of genetic data and idioms to think about social identities: the identity of *mestiço*, which is deeply racialized, is validated in genetic terms, even as race itself and racial differences are denied.

This re-racialization is evident in the use in the public domain of notions such as ‘black genes’ or ‘white ancestry’, which conflate genetic ancestry with racial identity. When some anti-quota commentators claimed that Neguinho da Beija-Flor or black activists were ‘not black’ or even ‘white’ on the basis of European ancestry, or when *Globo* coined the term ‘Brazipeans’, they were using genetics to redefine identities in racial terms. Here, genetics is deployed to speak about race and to arbitrate racial identity. It is also used to privilege European heritage, a notion closely articulated with whiteness in Brazil. This serves less to de-racialize Brazilian society than to de-Africanize its population. Genetics effectively offers new avenues for whitening individual and collective identities. The important distinction made by geneticists and social scientists between ancestry and social identity is lost to many participants and audiences of the affirmative action debate, who partly understand race in genetic terms.

We say ‘re-geneticize’ here because the data introduced by recent genomic techniques did not produce brand-new configurations of race and nation or biology and society, but re-worked established themes about the importance or unimportance of race, in which genetics had played a role over previous decades, as it explored racial mixture using older technologies (De Souza and Santos, 2014). The articulation of genetic data with the notions of racial democracy, pervasive mixture and a unified Brazilian identity that were already circulating prominently before and during the debate on affirmative action is a crucial vehicle for the wider dissemination of these data and their transformation into a political weapon. Genetic data play a greater part in public debates about these themes than at any time since the era of eugenics. This imbues genetics with social and political currency, and grants scientific support to pre-existing discourses through their translation into a genetic idiom. The popular usage of genetics in the form of black-boxed and formulaic anecdotes, rather than through discussions of scientific detail, significantly contributes to the reproduction of such discourses instead of transforming everyday racial conceptions in a fundamental way. The tensions between mixture and whitening that run through dominant forms of thinking about national identity in Brazil have resurfaced in the political uses of genetic data and arguments, which are used simultaneously to re-articulate the ideals of mixture and racial democracy, and to Europeanize both black people and the Brazilian population as a whole.

The political implications of the uses of genetic data are similarly uneven. Many anti-quota social scientists who make reference to genetic arguments espouse a progressive social agenda (e.g. Peter Fry, Ricardo Ventura Santos, Marcos Chor Maio). They do not deny racial inequality or racism in Brazil, but for them solutions are found in progressive social policies addressing class inequality in general. People on the far left criticize racial quotas on similar grounds. In contrast, there is little trace of such a challenge to either the status quo or racial thinking in the positivistic way in which a *Globo* editorial affirmed that science has proven the nonexistence of Afro-Brazilians. Rather the editorial sought to settle by reference to scientific authority the question of the nation’s identity. Thus, the political use of genetics can go either way: it may deconstruct race, with a class agenda in mind, and it can re-essentialize a unified *mestiço* identity, while also privileging European ancestry and whiteness. This said, it is clear that, in Brazil, genetic data have been incorporated most publicly into the affirmative action debate first by those who criticize affirmative action from diverse political perspectives and, second, by mass media commentators who propound particular political arguments about the irrelevance of racial difference and the Europeanized mixedness of the Brazilian population. The alignment of genetics with the vested interests of quite powerful political players and the mass media is key to the arguments’ widespread dissemination.

The political outcome of the affirmative action debate is instructive for thinking about the role genetic knowledge has to play in questions of citizenship and social policy. The genetic data showing that the Brazilian population is mixed could align quite nicely with images of the nation as racially unified, without a separate black population and as, if anything, more European than African. In that sense, the genetic data could be deployed to resonate with a mid-20th-century status quo, reinvigorating for Brazil today a viewpoint from which racial dimensions of unequal social relations are de-historicized and made invisible. Underwriting this, the framing of genetics as objective and apolitical made it into an ‘anti-politics machine’ (Ferguson, 1990), even as genetic data were being used in overtly political arguments. Genetics is deployed by some to take questions of race and racism away from the sphere of politics and towards a technical discussion about the (im)possibility of classification. The apparent aim is to shift the focus from social inequality to biological difference, resulting in some cases in a conflation of sameness with equality as part of arguments that deny the necessity of race-based affirmative action.

Yet, in the end, the Supreme Court, the government and the state stuck with the post-1990s agenda of state multiculturalism, the official recognition of a black population disadvantaged by racism, and the appropriateness for Brazil of racially differentiated social policies. The 2012 law retained quotas for poorer people, but also for black people. The Court went with the arguments of the proponents of racial quotas – whether in the black social movement, social science or the state multiculturalist bureaucracy – that genetics was irrelevant when it came to affirmative action in the social domain. Citizenship entitlements, in terms of access to education and similar domains, should be decided on social criteria, not genetic ones. Interestingly, although the detail of this cannot be covered here, in relation to race-based policies related to access to health care, the state was more attentive to genetic arguments (see Fry, 2005b; Kent et al., 2014; Maio and Monteiro, 2005). The fact that white Brazilians could carry genetic variants related to sickle-cell anaemia meant that the image of the condition as a ‘black disease’, which was being promoted by some in the black movement, did not stick, and screening for these variants was not restricted to black people. In short, the state’s response was varied according to the perceived relevance of genetic arguments.

The Brazilian material presented here shows just how uneven and indeed contradictory the effects of genetic knowledge can be, as it circulates across the varied topography of the ideological and political terrain that it itself helps to shape. Social science research that enquires into the impact of genetic knowledge on social relations and social divisions can benefit from an appreciation of the diverse and inconsistent ways genetics does and does not gain traction in different sectors of society and in relation to different political standpoints.

## Notes

1. According to project coordinator Silvia Salek (Gaspar Neto et al., 2012).

2. In addition, the African and Amerindian contribution to his DNA were, respectively, 31.5 percent and 1.4 percent.

3. Interview with Kaufmann in magazine *Istoé*. Available at: http://www.terra.com.br/istoe-temp/edicoes/2012/artigo89658-1.htm (accessed 25 September 2014.)

4. See http://www.bbc.co.uk/portuguese/reporterbbc/story/2007/05/070424_dna_neguinho_cg.shtml (accessed 25 September 2014.)

5. See http://www.bbc.co.uk/portuguese/reporterbbc/story/2007/05/070507_dna_freidavid_cg.shtml (accessed 25 September 2014.)

6. The term *movimento negro* in Brazil encompasses a multiplicity of entities, with diverse agendas and strategies, which episodically collectively rally around specific issues, such as racial quotas (Alberti and Pereira, 2006). Here, we refer to the black movement as shorthand for the multiple actors involved in the debate, without intending to suggest homogeneity.

7. See, for example, Duster (2006), Fujimura and Rajagopalan (2011), Fullwiley (2007), M’Charek (2005), Montoya (2011), Nash (2012), Reardon (2005) and Wade et al. (2014).

8. For example, Kent (2013), M’Charek (2013), Nelson (2008, 2013), Reardon and TallBear (2012), Schramm et al. (2012) and Wailoo et al. (2012).

9. Geneticists working at other universities have approached the study of the Brazilian population differently, often placing stronger emphasis on regional and ethnic-racial variation (Kent et al., 2014). However, such studies have not received the same public coverage as Pena’s research.

10. Unless otherwise stated, quotations are derived from interviews with Kent or from his field observations. All translations from Portuguese are by Kent.

11. See the special issues of the following journals, which include articles by Pena and other geneticists, and show the diversity of social science opinion, with social scientists such as Livio Sansone, José Jorge de Carvalho, José Carlos dos Anjos, Kabengele Munanga, Rita Segato and Antonio Sérgio Guimarães supporting racial quotas: *Horizontes Antropológicos*, 2005, 11(23); *Revista USP*, 2005, 68; and, *Estudos Avançados*, 2004, 18.

12. The website of Pena’s commercial laboratory – Laboratorio Gene – features some 200 media items about his research, many of which relate to race and quotas (Gaspar Neto et al., 2012).

13. For example, the Yahoo Brazil discussion group on racial discrimination featured dozens of messages on the subject in the days after the dissemination of the Afro-Brazilian roots project (http://br.dir.groups.yahoo.com/group/discriminacaoracial/, consulted 25 September 2014).

14. http://br.groups.yahoo.com/group/discriminacaoracial/message/33847, consulted 25 September 2014.
